# Reactions of *Plasmodium falciparum* Type II NADH: Ubiquinone Oxidoreductase with Nonphysiological Quinoidal and Nitroaromatic Oxidants

**DOI:** 10.3390/ijms26062509

**Published:** 2025-03-11

**Authors:** Lina Misevičienė, Marie-Pierre Golinelli-Cohen, Visvaldas Kairys, Audronė Marozienė, Mindaugas Lesanavičius, Narimantas Čėnas

**Affiliations:** 1 Department of Xenobiotics Biochemistry, Institute of Biochemistry of Vilnius University, Saulėtekio 7, LT-10257 Vilnius, Lithuania; lina.miseviciene@bchi.vu.lt (L.M.); audrone.maroziene@bchi.vu.lt (A.M.); mindaugas.lesanavicius@gmc.vu.lt (M.L.); 2 Institut de Chimie des Substances Naturelles, UPR2301, CNRS, Université Paris-Saclay, 1, Avenue de la Terrasse, 99198 Gif-sur-Yvette, France; marie-pierre.golinelli@cnrs.fr; 3 Department of Bioinformatics, Institute of Biotechnology of Vilnius University, Saulėtekio 7, LT-10257 Vilnius, Lithuania; visvaldas.kairys@bti.vu.lt

**Keywords:** type II NADH:ubiquinone oxidoreductase, *Plasmodium falciparum*, quinones, nitroaromatic compounds, reduction mechanism

## Abstract

In order to detail the antiplasmodial effects of quinones (Q) and nitroaromatic compounds (ArNO_2_), we investigated their reduction mechanism by *Plasmodium falciparum* flavoenzyme type II NADH:ubiquinone oxidoreductase (*Pf*NDH2). The reactivity of Q and ArNO_2_ (*n* = 29) follows a common trend and exhibits a parabolic dependence on their single-electron reduction potential ($E_{7}^{1}$), albeit with significantly scattered data. The reactivity of quinones with similar $E_{7}^{1}$ values increases with their lipophilicity. Quinones are reduced by *Pf*NDH2 in a two-electron way, but ArNO_2_ are reduced in a single-electron way. The inhibition studies using NAD^+^ and ADP-ribose showed that quinones oxidize the complexes of reduced enzyme with NADH and NAD^+^. This suggests that, as in the case of other NDH2s, quinones and the nicotinamide ring of NAD(H) bind at separate sites. A scheme of *Pf*NDH2 catalysis is proposed, consistent with both the observed ‘ping-pong’ mechanism and the presence of two substrate binding sites. Molecular docking showed that Q and ArNO_2_ bind in a similar manner and that lipophilic quinones have a higher affinity for the binding site. One may expect that *Pf*NDH2 can be partially responsible for the previously observed enhanced antiplasmodial activity of aziridinylbenzoquinones caused by their two-electron reduction, as well as for the redox cycling and oxidative stress-type action of ArNO_2_.

## 1. Introduction

Although antimalarial drugs have successfully mitigated the epidemics in the past few decades, malaria still remains a major challenge to global health. The emergence of the resistance of the malaria parasite *Plasmodium falciparum* to available drugs (e.g., artemisinin, chloroquine and other aminoquinoline derivatives, amino alcohols [1,2]) has resulted in the demand for new antimalarial agents, for a better understanding of their mechanisms of action, and also for the identification of new drug targets. Since *P. falciparum* has a weaker antioxidant defense than the host cells, considerable attention is paid to prooxidant and/or redox cycling compounds, and efforts are also being made to identify and characterize the enzymes responsible for their reductive activation [3,4,5,6,7,8,9,10]. From this point of view, flavoenzymes of various classes are valuable due to the wide spectrum of compounds they reduce, but information on this issue is limited [3].

The mitochondrial electron transport chain of *Plasmodium falciparum* lacks the normal higher animal proton-pumping NADH:ubiquinone oxidoreductase (Complex I). Instead, they have an alternative type II flavin adenine dinucleotide (FAD)-containing NADH: ubiquinone oxidoreductase (*Pf*NDH2) [11,12,13,14] that belongs to a different type of enzymes commonly found in bacterial, fungal, and plant mitochondria but not in mammalian mitochondria [15,16,17,18,19,20,21,22,23,24,25]. These enzymes are composed of a single polypeptide chain and lack a transmembrane domain. Another distinguishing feature is that the NADH nicotinamide ring and ubiquinone bind in separate sites at the isoalloxazine ring of the flavin [18,20]. This naturally made *Pf*NDH2 a target for potential antiplasmodial agents. Indeed, the antiplasmodial activity of the quinolones CK-2-68 and RYL-552 and their analogues was attributed to their effective inhibition of *Pf*NDH2, which was also characterized by X-ray structural analysis of the enzyme–inhibitor complex [14]. However, subsequent studies made this view debatable, as the antiplasmodial effect of these compounds was attributed to the inhibition of the parasite cytochrome *bc*_1_ complex [26].

On the other hand, *Pf*NDH2 may have properties linked to the action of redox active antiplasmodial agents. The redox active antiparasitic agents such as quinones, nitroaromatic compounds, isoalloxazines, and aromatic *N*-oxides may be reduced by flavoenzymes or by their redox partners, e.g., Fe-S proteins, in a single- or two-electron way [27,28,29,30,31]. Free radicals of compounds formed during one-electron reduction can undergo redox cycling and cause oxidative stress. During the two(four)-electron reduction of nitroaromatic compounds, DNA-alkylating hydroxylamines are formed [30,31]. Two-electron reduction of aziridinyl-substituted quinones leads to the formation of DNA-alkylating aziridinyl-hydroquinones [32]. However, in the case of *P. falciparum* flavoenzymes, these reactions have been studied in detail only for ferredoxin:NADP^+^ oxidoreductase [5,6,27] and for NADPH:glutathione- and thioredoxin reductases [33], while data on *Pf*NDH2 are scarce and fragmentary [34].

In this pilot study, we characterized the mechanism of reduction of quinones and nitroaromatics by *Pf*NDH2 and revealed some previously uncharacterized details of the mechanism of its catalysis, which may be important for understanding the functioning of other NDH2s. Studies of a broad range of oxidants have shown that their reactivity is determined by their reduction potential and lipophilicity. The potential role of the enzyme on the in vitro antiplasmodial activity of these classes of prooxidant compounds is discussed.

## 2. Results

### 2.1. Steady-State Reactions of PfNDH2

**Figure 1:**
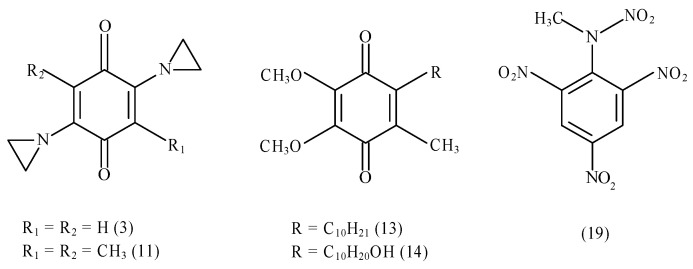
The structures of nontrivial oxidants used in this study. The numbers of compounds correspond to those given in Table 1: DZQ (**3**), MeDZQ (**11**), decylubiquinone (**13**), idebenone (**14**), and tetryl (**19**).

In order to determine the influence of charge transfer energy on the reactivity of *Pf*NDH2 with oxidants, a series of quinones (Q) and nitroaromatic compounds (ArNO_2_) with different single-electron reduction midpoint potentials ($E_{7}^{1}$) were investigated. The structures of nontrivial oxidants are presented in Figure 1. Among them, ubiquinone analogs decylubiquinone and idebenone, which have long-chain aliphatic substituents, can be noted.

The bimolecular rate constants (*k*_cat_/*K*_m_) for the reduction of these compounds and their maximal reduction rate constants ${(k}_{\mathrm{cat}}^{\mathrm{app}}),$ determined at a fixed concentration of NADH, 100 µM, are presented in Table 1.

**Figure 2:**
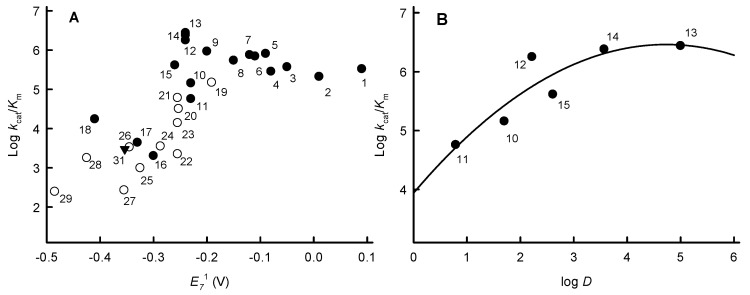
(**A**) Relationship between the log *k*_cat_/*K*_m_ of quinones (solid circles) and nitroaromatics (blank circles) and their single-electron reduction midpoint potentials at pH 7.0 ($E_{7}^{1}$). Numbers and reduction potentials of compounds are given in Table 1. The log *k*_cat_/*K*_m_ of the single-electron oxidant benzylviologen (31) is shown as an inverted triangle. (**B**) Relationship between the log *k*_cat_/*K*_m_ of quinones with $E_{7}^{1}$ = −0.23–−0.26 V and their octanol/water distribution coefficient at pH 7.0 (log *D*).

Figure 2A shows that the reactivity (log *k*_cat_/*K*_m_) of Q and ArNO_2_ follows a common trend and exhibits a parabolic (quadratic) dependence on their $E_{7}^{1}$, reaching the maximal values at $E_{7}^{1}$ ≅ −0.25 V. However, the data are scattered and regression is characterized by *r*^2^ = 0.5739 and F(2.26) = 17.51. On the other hand, the reactivity of a series of benzoquinones with similar $E_{7}^{1}$ values, −0.23 V to −0.26 V, was found to increase with increasing lipophilicity (log *D*) (Figure 2B). Introducing log *D* as an additional correlation parameter improves the overall regression (compounds **1**–**29**) only slightly, giving *r*^2^ = 0.6065 and F(3.25) = 12.68.

**Figure 3:**
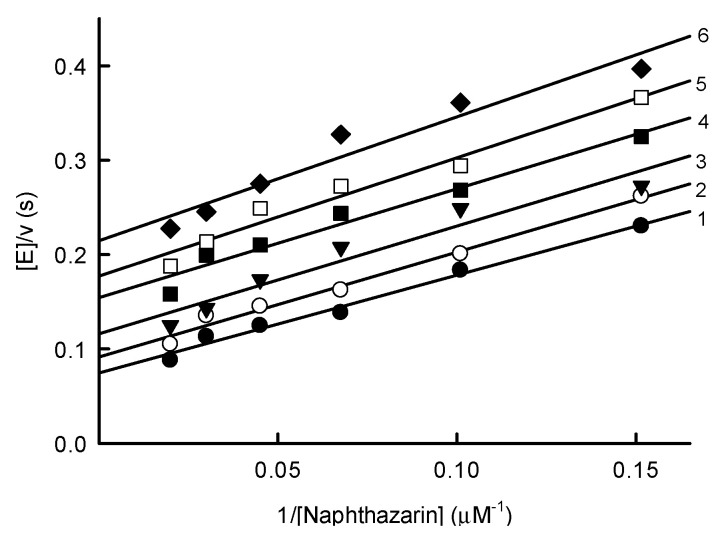
Steady-state kinetics of a reduction of naphthazarin by NADH catalyzed by *Pf*NDH2 at varied NADH concentrations. NADH concentrations: 100 μM (1), 50 μM (2), 25 μM (3), 16.7 μM (4), 12.5 μM (5), and 8.5 µM (6).

More detailed studies were performed using one of the most active *Pf*NDH2 oxidants, 5,8-dihydroxy-1,4-naphthoquinone (naphthazarin). Varying the concentration of naphthazarin at several fixed concentrations of NADH showed that the reaction rate was described by a series of parallel lines in Lineweaver–Burk coordinates (Figure 3). This indicates that the kinetics of *Pf*NDH2 follow a ‘ping-pong’ mechanism. In this case, the *k*_cat_ of the enzyme calculated at infinite NADH concentration is equal to 16.2 ± 0.2 s^−1^ and NADH *k*_cat_/*K*_m_ is equal to 1.0 ± 0.1 × 10^6^ M^−1^s^−1^.

**Figure 4:**
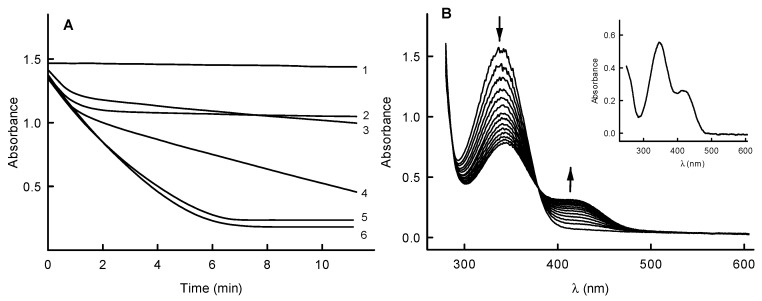
(**A**) Kinetics of oxidation of 250 µM NADH by 50 nM *Pf*NDH2 in the absence of oxidant (1) and in the presence of 50 µM 2,6-dimethyl-1,4-benzoquinone at pH 7.0 (2), 50 µM 1,4-naphthoquinone at pH 5.5 (3) or pH 7.0 (4), and 50 µM naphthazarin at pH 5.5 (5) or pH 7.0 (6). (**B**) Formation of *N*-methylpicramide during the oxidation of 250 µM NADH by 50 µM tetryl in the presence of 50 nM *Pf*NDH2. The arrows show the decrease in absorbance at 340 nm due to NADH oxidation and absorbance increase at 420 nm. The spectra are recorded every 3 min. Inset shows absorbance spectrum of 50 µM *N*-methylpicramide.

At pH 7.0 and pH 5.5, naphthazarin oxidizes a significant excess of NADH in an apparently monophasic process (Figure 4A). However, using another oxidant, 1,4-naphthoquinone, the biphasic nature of the process becomes evident, i.e., the reaction rate slows down after oxidizing a stoichiometric amount of NADH. The biphasic nature of this reaction is more pronounced at pH 5.5, while 2,6-dimethyl-1,4-benzoquinone at pH 7.0 oxidizes only a stoichiometric amount of NADH (Figure 4A). Decilubiquinone and idebenone also oxidize only stoichiometric amounts of NADH. This suggests that *Pf*NDH2 performs a two-electron reduction of quinones, because the second oxidation phase is limited by the rate of reoxidation of two-electron reduced 1,4-naphthoquinone and benzoquinone derivatives by oxygen, which is additionally slowed down at lower pH [37,38]. In contrast, the hydroxy-substituted naphthohydroquinones are rapidly oxidized at neutral pH [37,39]. This explains why hydroxy-substituted naphthoquinones oxidize excess NADH in a single phase (Figure 4A). Quantitatively, the single- and two-electron reduction of quinones by NAD(P)H-oxidizing flavoenzymes can be measured using the acceptor 1,4-benzoquinone, since at pH ≤ 7.0, its semiquinone reduces cytochrome *c* rapidly (*k* ~ 10^6^ M^−1^s^−1^) while its two-electron reduction product, hydroquinone, reduces cytochrome *c* at a negligible rate [40]. During *Pf*NDH2-catalyzed oxidation of NADH by 1,4-benzoquinone at pH 7.0, the reduction of added cytochrome *c* took place with less than 10% of NADH oxidation rate. In this case, the single-electron flux in the reduction of quinones expressed as the ratio between the rate of cytochrome *c* reduction and doubled oxidation rate of NADH [40] is equal to 5%.

When examining the nitroreductase activity of *Pf*NDH2, 2,4,6-trinitrophenyl-*N*-methylnitramine (tetryl) was observed to oxidize a significant excess of NADH (Figure 4B). At the end of the reaction, the product spectrum corresponds to that of *N*-methylpicramide with characteristic absorption at 340 nm and 420 nm (Figure 4B, inset). The reaction is accompanied by reduction of cytochrome *c* (50 µM) added to the reaction medium, which occurs at a rate of 190 ± 5% compared to NADH oxidation. Superoxide dismutase (100 U/mL) inhibits cytochrome *c* reduction by 70–80%. At the end of the reaction, 45 ± 3 µM nitrite is formed from 50 µM tetryl. This indicates that *Pf*NDH2 performs reductive denitration of tetryl in a single-electron way, forming an intermediate tetryl anion-radical, which participates in redox cycling [41]. During the reduction of TNT or *p*-dinitrobenzene, reduction of added cytochrome *c* also occurs with 190–200% of the rate of NADH oxidation. These reactions are also inhibited by 100 U/mL superoxide dismutase by 70–80%. Taken together, this suggests that unlike the two-electron reduction of quinones, *Pf*NDH2 performs the single-electron reduction of ArNO_2_.

### 2.2. Inhibition Studies of PfNDH2

**Figure 5:**
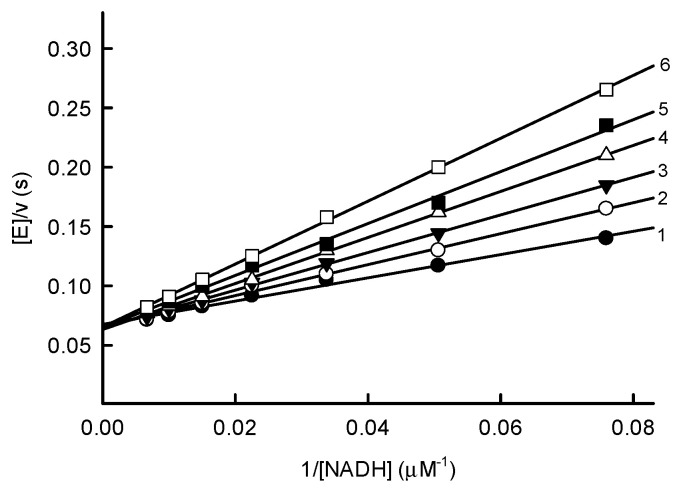
NAD^+^ as a competitive to NADH inhibitor in *Pf*NDH2-catalyzed reactions in the presence of 50 µM naphthazarin: 1—no NAD^+^, 2—1.25 mM NAD^+^, 3—2.5 mM NAD^+^, 4—3.75 mM NAD^+^, 5—5.0 mM NAD^+^, 6—7.5 mM NAD^+^.

While analyzing the mechanisms of action of NDH2 from various sources, inhibition by the reaction product NAD^+^ was also studied [15,17,19]. However, the obtained data are fragmentary and mainly concerned the competition of NAD^+^ with NADH, the former binding to the oxidized form of the enzyme [19]. We found that at high concentrations of naphthazarin, an order of magnitude above its *K*_m_, NAD^+^ is a competitive inhibitor for NADH with *K*_i_ = 3.7 ± 0.4 mM (Figure 5). ADP-ribose, lacking the nicotinamide ring, also acts as a competitive inhibitor with *K*_i_ = 2.5 ± 0.3 mM. These *K*_i_ describe the binding of NAD^+^ and ADP-ribose to the oxidized form of the enzyme. Importantly, the inhibition character of these compounds was linear, i.e., a linear dependence of reciprocal *k*_cat_/*K*_m_ of NADH on inhibitor concentration in Cleland coordinates was observed.

**Figure 6:**
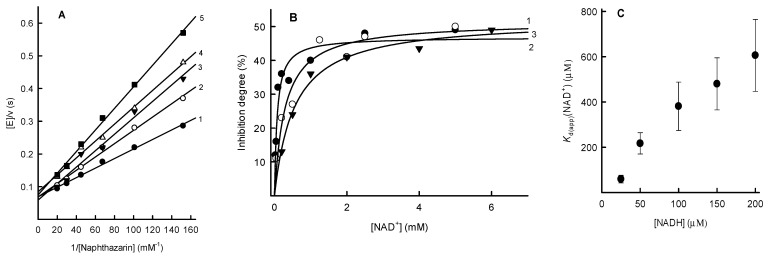
NAD^+^ as an inhibitor towards naphthazarin in the PfNDH2-catalyzed reaction. (**A**) Competitive inhibition of NAD^+^ towards naphthazarin in the presence of 200 µM NADH: 1—no NAD^+^, 2—0.6 mM NAD^+^, 3—1.0 mM NAD^+^, 4—2.0 mM NAD^+^, 5—6.0 mM NAD^+^. (**B**) Dependence of inhibition efficiency on NAD^+^ concentration at 200 μM (1), 50 μM (2), and 25 μM (3) NADH. (**C**) Dependence of the calculated NAD^+^ binding constants (*K*_d(app)_(NAD^+^)) on NADH concentration.

At [NADH] >> *K*_m_, NAD^+^ also acts as a competitive inhibitor towards naphthazarin (Figure 6A). Importantly, however, in this case, the inhibition is incomplete, i.e., at high NAD^+^ concentrations, the *k*_cat_/*K*_m_ of naphthazarin decreases to a limiting value close to 50% (Figure 6B). At lower NADH concentrations, the efficiency of NAD^+^ inhibition increases, but the maximum degree of inhibition remains the same. Calculated NAD^+^ binding constants (*K*_d(app)_(NAD^+^)) increase linearly with NADH concentration (Figure 6C).

This indicates that NADH and NAD^+^ compete for the same binding site in the reduced enzyme form. However, due to the modest maximal inhibitory effect of NAD^+^, 50%, the *K*_d_ of NAD^+^ and NADH complexes cannot be precisely determined in this case. They can be expected to be in the lower micromolar range (Figure 6C). A similar maximum degree of inhibition by NAD^+^, 2.1 times, is observed for *k*_cat_/*K*_m_ of idebenone, but for TNT, it is equal to 3.0. In contrast, in the presence of 250 µM NADH, ADP-ribose acts as a weak uncompetitive inhibitor for naphthazarin (*K*_i_ = 13.7 ± 2.3 mM), i.e., it does not decrease the *k*_cat_/*K*_m_ of naphthazarin.

### 2.3. Molecular Modeling of Oxidant Binding in the Active Center of PfNDH2

In order to detail the mechanism of action of *Pf*NDH2 and the influence of the structure of the substrates on its specificity (Figure 2A,B), molecular modeling of their binding was performed using the GNINA docking program. GNINA, which is derived from Autodock Vina [42], utilizes an ensemble of convolutional neural networks (CNNs) as a scoring function and shows a marked scoring accuracy improvement [43].

Kinetic studies (Figure 6) showed that the oxidants oxidize the complexes of the reduced enzyme with NAD^+^ and NADH. Therefore, the structure of *Pf*NDH2 with bound NAD(H) (PDB id: 5JWB) [14] was chosen for analysis, focusing on the ability of the oxidant to form an H-bond with isoalloxazine N3H and its ability to occupy the binding site of the quinone ring. In this structure, the isoalloxazine ring of FAD has been converted to its reduced form for adequate visualization of the reduction pathway [44,45]. For docking validation, we used the structure of the ternary complex of the structurally similar *S. cerevisiae* Ndi1 with NAD(H) and ubiquinone (PDB id: 4G73) [18]. In this case, ubiquinone was docked into the active sites of both enzymes, Ndi1 and *Pf*NDH2 (Supplementary Materials S1).

**Figure 7:**
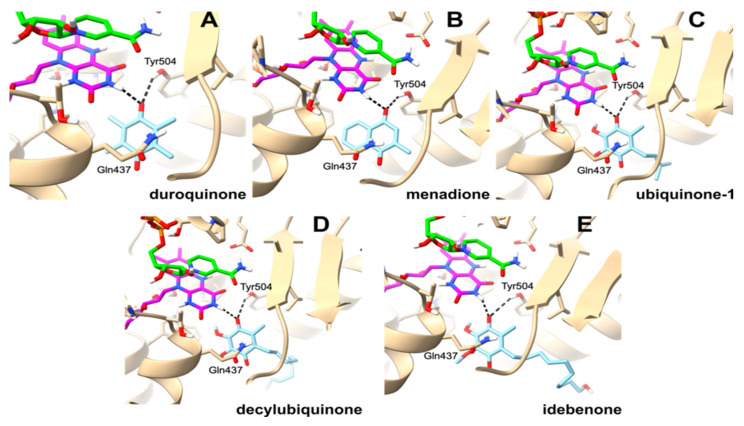
Docked conformations of quinones in the active site of *Pf*NDH2: (**A**) duroquinone; (**B**) menadione; (**C**) ubiquinone-1; (**D**) decylubiquinone; (**E**) idebenone. FAD is colored magenta, NAD(H) is colored green, quinone is colored light blue. Hydrogen bonds are shown as dotted lines.

**Figure 8:**
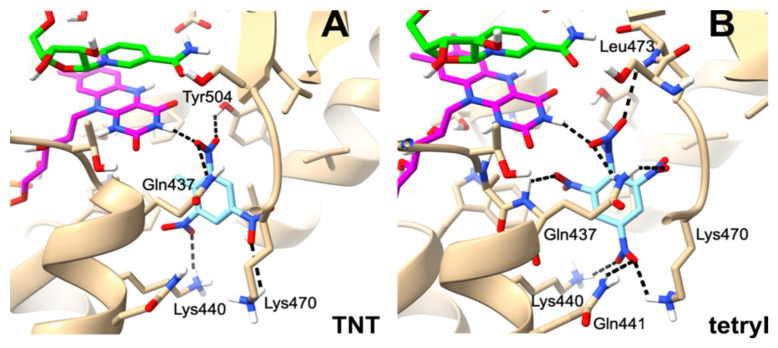
Docked conformations of nitroaromatic compounds in the active site of *Pf*NDH2: (**A**) TNT; (**B**) tetryl. FAD is colored magenta, NAD(H) is colored green, nitroaromatic compound is colored light blue. Hydrogen bonds are shown as dotted lines.

The obtained docking data show that quinones bind at the *si*-side of the isoalloxazine, and that apart from the formation of the H-bond with its N3H, the carbonyl groups of quinones form H-bonds with Tyr504, and the quinone ring interacts with Gln437 (Figure 7). The chains of the long aliphatic substituents of decylubiquinone and idebenone extend outside the isoalloxazine environment, with idebenone hydroxyl forming an H-bond with Gln506. In the case of binding of nitroaromatic compounds, the 2(6)-nitro group of TNT and the nitro group of the tetryl *N*-nitramine substituent form H-bonds with isoalloxazine N3H (Figure 8). These compounds also form H-bonds with Gln437. It should be noted that the studied ArNO_2_ form more H-bonds than quinones: in addition to Tyr504 (or Leu473 in the case of tetryl), Lys440, Lys470, and Gln441 are involved in the interaction (Figure 8). The full list of all ligand contacts with the protein residues and their interaction areas is presented in Table S1 (Supplementary Materials).

The compound binding affinities calculated using the CNN scoring method with the GNINA program [43], expressed as kJ/mol, are equal to −23.76 (menadione), −24.31 (duroquinone), −28.87 (ubiquinone (Q1)), −32.68 (decylubiquinone), −32.97 (idebenone), −25.36 (TNT), and −28.32 (tetryl). This shows that the lipophilic decylubiquinone and idebenone, which are more reactive than duroquinone with similar electron accepting properties (Table 1, Figure 2B), also have a higher affinity for the active site.

## 3. Discussion

**Scheme 1:**
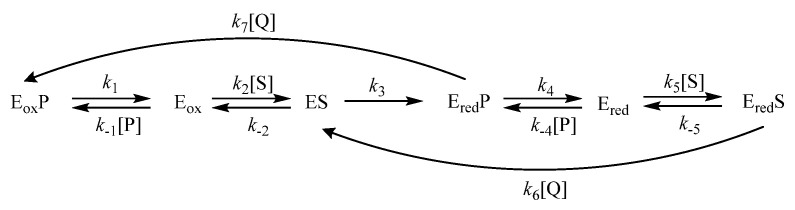
The proposed mechanism of quinone reductase reaction of *Pf*NDH2.

In this study, we obtained information relevant to the catalysis of *Pf*NDH2 and to other type-II NDHs. We also obtained background information that may be relevant for the development of redox-active antiplasmodial agents targeting *Pf*NDH2. One of the main problems in catalysis of type-II NDHs is an apparent contradiction between the ‘ping-pong’ mechanism (a series of parallel lines in double reciprocal coordinates, Figure 3) with two separate binding sites of NADH and quinones, which implies the formation of a ternary complex [18,20,22]. A similar case, the ‘hybrid ping-pong’ mechanism, is known for glutathione reductase, when glutathione oxidizes the free reduced enzyme and its complexes with NADPH and NADP^+^ at similar rates [46]. Unlike in *Mycobacterium tuberculosis* NDH2, where NAD^+^ acts as an uncompetitive inhibitor for quinone and does not change its *k*_cat_/*K*_m_ [19], we found that NADH and NAD^+^ compete for binding to the reduced *Pf*NDH2, and that the quinone oxidizes the complex with NAD^+^ about twice more slowly than its complex with NADH (Figure 6). The presented simplified mechanism of *Pf*NDH2 (Scheme 1) is similar to our previous one for NADPH:thioredoxin reductase from *Arabidopsis thaliana* [47].

In this scheme, E_ox_, E_red_, ES, E_red_P, E_red_S, and E_ox_P are the oxidized enzyme, reduced enzyme, enzyme–substrate complex, complex of reduced enzyme with NAD^+^, complex of reduced enzyme with NADH, and the complex of the oxidized enzyme with NAD^+^, respectively, and S, P, Q are NADP, NAD^+^, and quinone. We assume that NAD^+^ does not reoxidize the reduced enzyme, because analogous type-II NDH from *Staphylococcus aureus* and *Escherichia coli* possess the standard redox potentials of −0.220 V [21,48], i.e., much higher than the redox potential of NAD/NADH, −0.320 V. Further, we assume that the redox forms E_red_, E_red_P, and E_red_S are in rapid equilibrium, and that at high S and/or P concentrations, [E_red_] ≅ 0. With the help of Cha’s simplification [49], the resulting rate Equation (1) is expressed using the equilibrium constants *K*_1_ = *k*_1_/*k*_−1_, *K*_4_ = *k*_4_/*k*_−4_, and *K*_5_ = *k*_−5_/*k*_5_, which are the *K*_d_ of complexes E_ox_P, E_red_P, and E_red_S, respectively:

(1) $$\frac{\left[E\right]}{v}=\frac{1}{k_{3}}\left(1+\frac{k_{-2}}{k_{2}\left[S\right]}\left(1+\frac{\left[P\right]}{K_{1}}\right)\right)+\frac{K_{5}\left[P\right]+K_{4}\left[S\right]}{k_{7}\left[P\right]K_{5}+k_{6}\left[S\right]K_{4}}\left(\frac{1}{\left[Q\right]}+\frac{k_{7}\left[P\right]K_{5}}{k_{2}\left[S\right]\left(K_{5}\left[P\right]+K_{4}\left[S\right]\right)}\left(1+\frac{\left[P\right]}{K_{1}}\right)\right)$$

The derivation of this equation is detailed in Supplementary Materials (S2, Schemes S1 and S2). It is evident that Equation (1) is consistent with a decrease in *k*_cat_/*K*_m_ of NADH in the presence of NAD^+^ (Figure 5) and the dependence of *k*_cat_/*K*_m_ of quinone on NAD^+^/NADH ratio (Figure 6). The contradiction between ternary complex formation during catalysis of NDH2 from different sources and ‘ping-pong’ patterns of reaction ([18,20,22], and Figure 3 of the present work) is explained by the transformation of Equation (1) into Equation (2) in the absence of NAD^+^ ([P] = 0), the latter corresponding to the ‘ping-pong’ mechanism:

(2) $$\frac{\left[E\right]}{v}=\frac{1}{k_{3}}+\frac{k_{-2}}{k_{2}k_{3}\left[S\right]}+\frac{1}{k_{6}\left[Q\right]}$$

Therefore, we expect that this information may also be valuable in characterizing the mechanisms of catalysis of other NDH2s.

Regarding the further analogies between *Pf*NDH2 and other type-II NDHs, it should be noted that the uncompetitive inhibition of ADP-ribose with respect to quinone is analogous to the effects of AMP lacking a nicotinamide ring on *S. cerevisiae* NDH2 [15]. The slight inhibitory effect of NAD^+^ (Figure 6A,B) can be related not only to the shielding of the isoalloxazine ring but also to the decrease in the electron density on the reduced isoalloxazine due to partial charge transfer to NAD^+^ [50]. A somewhat similar phenomenon was observed in *Caldalkalibacillus thermarum* NDH2, where quinone oxidizes the complex of the reduced enzyme with NAD^+^ about 1.5 times slower than the free enzyme [22]. Our estimated micromolar *K*_d_ of the complex of NAD^+^ and reduced *Pf*NDH2 (Figure 6C) is in the range of the calculated *K*_d_ for other NDH2s, <2 µM (*C. thermarum*) [22] and 10–20 µM (*S. aureus*) [21]. In addition, different $k_{\mathrm{cat}}^{\mathrm{app}}$ values for various oxidants (Table 1) show that the limiting step of the *Pf*NDH2 catalytic cycle is an oxidative half-reaction, as in the case of other type-II NDHs [21,22].

In *Pf*NDH2-catalyzed reduction, quinones and ArNO_2_ do not form separate series of different reactivity (Figure 2A). The computer modeling data (Figure 7 and Figure 8) show that these compounds bind in the same domain with the participation of the same amino acids, Gln437 and Gln441. Similar motifs, Gln394, Gln398, and Gln317, Gln321 are found in the quinone binding sites of yeast Ndi1 (PDB id: 4G73) and *C. thermarum* NDH2, respectively [18,20]. However, the common dependence of the reactivity of quinones and ArNO_2_ on $E_{7}^{1}$ is strongly scattered (Figure 2A). This shows a significant impact of the structure of oxidants on their reactivity, the factors of which will be the subject of our further studies. However, there exists a linear correlation (*r*^2^ = 0.9216) between the calculated quinone binding affinities and quinone *k*_cat_/*K*_m_ (Table 1). This demonstrates that docking could be used to design new ligands with better catalytic properties. Currently, it can be stated that the reactivity of quinones is determined to a significant extent by their lipophilicity (Figure 2B), which in turn can be linked to the lipophilicity of their binding site [18,20]. Analogously, more lipophilic homologous quinones were more reactive with *M. tuberculosis* NDH2 [19].

Another important point is that *Pf*NDH2 reduces quinones in a two-electron way, while ArNO_2_—in a single-electron way (Figure 4A,B). The increase in the rate of single-electron reduction of ArNO_2_ with an increase in their $E_{7}^{1}$ (Figure 2A) is formally consistent with the outer-sphere electron transfer model with low interaction between the electronic systems of the reactants [27,51,52]. The low degree of interaction of the electronic systems of Q and ArNO_2_ with isoalloxazine can be seen from Figure 7 and Figure 8 and is supported by the X-ray analysis of other type-II NDHs [18,20]. However, this makes the factors determining the two-electron reduction of quinones poorly understandable, as it is, as a rule, linked to the π-π interaction between the aromatic systems of the reactants [53] or to the proximity of the quinone carbonyl group to N5 of isoalloxazine [45]. On the other hand, there are assumptions about *Pf*NDH2 global motion during the catalytic cycle, which may affect electron/hydride transfer distances [54]. Assuming this, one of the possible explanations is that the reduction of both ArNO_2_ and quinones occurs with an initial single-electron transfer step, and that quinones are reduced in a three-step (e^−^, H^+^, e^−^) hydride transfer. The rate of reduction of quinones in this case also increases with their $E_{7}^{1}$ if the rate-limiting step is first electron transfer [36]. The following step, proton transfer, can be slowed down for nitroaromatic compounds due to the weaker basicity of their anion radicals (pK_a_ = 2–3) than most quinone radicals (pK_a_ = 4–5) [36,51]. This would allow ArNO_2_^•−^ to dissociate from the active site immediately after formation.

The current status of antiplasmodial prooxidant compounds, including quinones and nitroaromatics, is reviewed in [3] and partially in previous reviews [31,51]. Despite the abundance of data on their activity, the mechanisms and kinetics of single- or two-electron reduction by flavoenzymes of these compounds are not well studied. Exceptions here include *P. falciparum* ferredoxin:NADP^+^ oxidoreductase [5,6,27] and glutathione- and thioredoxin reductases [33]. Therefore, these data do not yet allow us to assess the role of individual flavoenzymes on the antiplasmodial activity of quinones and nitroaromatics, including the fact that their parallel mechanism of action, the inhibition of antioxidant disulfide reductases, may occur here [6,33]. However, based on our findings, some features of its catalysis could make *Pf*NDH2 an important target for redox-active antiplasmodial agents. In particular, it is the single-electron reduction of ArNO_2_ to their radicals, leading to their redox cycling and reactive oxygen species (ROS) formation. In terms of *k*_cat_/*K*_m_, the reactivity of ArNO_2_ towards *Pf*NDH2 (Table 1) is close to that of *Pf*FNR, the most active *P. falciparum* flavoenzyme responsible for the single-electron reduction of ArNO_2_ identified so far [6,27], thus implying that PfNDH2 and PfFNR may be of similar importance in this issue. However, because *P. falciparum,* during its intraerythrocyte stage, adopts microaerophilic metabolism and relies mainly on anaerobic processes [55,56], the role of the ROS-promoted parasite death should be interpreted with caution. On the other hand, microaerophilic conditions could contribute to the formation of other toxic ArNO_2_ reduction products, hydroxylamines [30,31,51].

Second, some aziridinyl-substituted benzo- and naphthoquinones were highly active against *P. falciparum* [4,5]. In the later case, their in vitro activity was two orders of magnitude higher than aziridine-unsubstituted quinones with the same redox potential [5]. An analogy can be drawn with the enhanced anticancer effects of aziridinyl-substituted quinones ([32], and references therein). In this case, in their two-electron reduction products (aziridinyl-hydroquinones), the electron-donating effect of -OH groups increases the reactivity of the aziridine ring with DNA. In mammalian cells, this reaction is carried out by the two-electron transferring NAD(P)H:quinone acceptor reductase (NQO1) [32]. We suggested that a net two-electron reduction of aziridinyl-benzoquinones to yield DNA-alkylating hydroquinones may take place in plasmodia, although two-electron transferring quinone reductases in *P. falciparum* have not been identified [5]. The data of this work show that the two-electron transferring *Pf*NDH2 possessing a considerable reactivity (Table 1) could be a potential candidate for the reductive activation of aziridinylbenzoquinones. Thus, part of the background information that may be relevant for the development of new redox-active antiplasmodial agents, our findings specifically highlight the potential importance of bioreductively activated quinones, e.g., substituted with aziridine or mustard groups. Due to the recent increasing interest in NDH2 from other sources, e.g., mycobacteria as potential drug targets [24,25], our data may also provide a new direction for their research, emphasizing their reactions with redox active agents.

## 4. Materials and Methods

### 4.1. Expression and Purification of PfNDH2

The open reading frame coding for *Pf*NDH2 (NCBI PF3D7_0915000) was inserted into a pET45 plasmid (Novagen, Sigma-Aldrich, St. Louis, MO, USA) that expressed the protein fused to a His-tag. The plasmid was used to transform *E. coli* Rosetta (DE3)pLys competent cells. Cell growth was performed at 37 °C in 6 L of Terrific Broth medium (Formedium, Norfolk, UK). At an OD_600_ of 0.8, the temperature was lowered to 18 °C, and protein overexpression was induced with 0.2 mM isopropyl β-D-1-thiogalactopyranoside (IPTG). Cells were incubated at 18 °C overnight and centrifuged. The purification was adapted from [34]. Cells were resuspended in 100 mL of 50 mM Hepes pH 8.0, 500 mM NaCl, 40 mM imidazole, 0.5% Triton X-100 completed with Complet antiprotease cocktail (Roche, Basel, Switzerland) and 5 mM β-mercaptoethanol. After sonication, cell extracts were centrifuged for 1 h at 30,000 rpm and 4 °C. The supernatant was loaded on a 5 mL HiTrap Chelating HP column (Cytiva, Marlborough, MA, USA) using an Akta Pure FPLC (Cytiva). The column was washed with buffer A (50 mM Hepes pH 8.0, 500 mM NaCl, 40 mM imidazole, 0.5% Triton X-100, 5 mM β-mercaptoethanol with a linear imidazole gradient (0–400 mM)) until NDH2 protein solution eluted. The fractions containing the protein were collected and concentrated. The buffer was exchanged into 50 mM Hepes pH 8.0 with 150 mM NaCl, 10% glycerol, and 0.1% Triton X-100. The protein was flash frozen and stored at −80 °C. For the determination of FAD content, enzyme samples were heat-denatured to liberate bound flavin, and its concentration in the resulting supernatants was measured according to έ_450_ = 11.3 mM^−1^cm^−1^.

### 4.2. Reagents and Enzymes

NAD(H), ADP-ribose, commercially available quinones, nitroaromatic compounds, and other reagents, horse heart cytochrome *c* and superoxide dismutase, were obtained from Sigma-Aldrich (St. Louis, MO, USA) and used as received. The following compounds were a generous gift of Dr. Jonas Šarlauskas (Institute of Biochemistry, Vilnius): aziridinyl-substituted quinones DZQ, MeDZQ and trimethyl-aziridinyl-1,4-benzoquinone (synthesized as described [5]), and 2,4.6-trinitrotoluene (TNT), tetryl and *N*-methylpicramide (synthesized as described in [57]).

### 4.3. Steady-State Kinetics Studies

The steady-state kinetics experiments were performed using a Cary60 UV/Vis spectrophotometer (Agilent Technologies, Santa Clara, CA, USA). All experiments were performed in 0.1 M Hepes buffer solution with 0.15 M NaCl and 1.0 mM EDTA at a pH of 7.0 and in certain cases, pH 5.5, at 25 °C. The stock solution of the enzyme (factor of dilution 1:100) was kept on ice in the same buffer solution containing 0.01% Triton X-100. The kinetic data were fitted to the Michaelis–Menten equation in SigmaPlot 14 (Systat Software, Inc., Chicago, IL, USA) (Equation (3)) to yield the steady-state parameters of the reactions, namely, the catalytic constants $k_{\mathrm{cat}}^{\mathrm{app}}$ and bimolecular reaction rate constants (or catalytic efficiency constants) *k*_cat_/*K*_m_ of the oxidants under a fixed concentration of NADH:

(3) $$\frac{v}{\left[E\right]}=\frac{k_{\mathrm{cat}}^{\mathrm{app}}\left[Q\right]}{K_{m}+\left[Q\right]}$$

where *v* is the reaction rate, [E] is the concentration of *Pf*NDH2, [Q] is the concentration of the oxidant, and $k_{\mathrm{cat}}^{\mathrm{app}}$ represents the number of molecules of NADPH oxidized by a single active site of the enzyme per second at saturated concentration of oxidant. The fitted parameters are equal to the reciprocal intercepts and slopes of Lineweaver–Burk plots, [E]/*v* vs. 1/[S], respectively. The kinetic parameters of the steady-state reactions according to the “ping-pong” mechanism were calculated according to Equation (4):

(4) $$\frac{v}{\left[E\right]}=\frac{k_{\mathrm{cat}}\left[Q\right]\left[\mathrm{NADH}\right]}{K_{m}^{\mathrm{NADH}}\left[Q\right]+K_{m}^{Q}\left[\mathrm{NADH}\right]+\left[Q\right]\left[\mathrm{NADH}\right]}$$

In this case, *k*_cat_ is the enzyme turnover number at saturating concentrations of both substrates. In the case of linear inhibition, the competitive inhibition constant (*K*_i_) of NAD^+^ or ADP-ribose ([I]) vs. NADH was calculated according to Equation (5):

(5) $$\frac{v}{\left[E\right]}=\frac{k_{\mathrm{cat}}^{\mathrm{app}}\left[\mathrm{NADH}\right]}{K_{m}^{\mathrm{NADH}}\left(1+\frac{\left[I\right]}{K_{i}}\right)+\left[\mathrm{NADH}\right]}$$

The uncompetitive inhibition constant of ADP-ribose vs. electron acceptor (Q) was calculated according to Equation (6):

(6) $$\frac{v}{\left[E\right]}=\frac{k_{\mathrm{cat}}^{\mathrm{app}}\left[Q\right]}{K_{m}^{Q}+\left[Q\right]\left(1+\frac{\left[I\right]}{K_{i}}\right)}$$

In the case of non-linear (incomplete) inhibition, *k*_cat_/*K*_m_ of quinone were determined separately for each NAD^+^ concentration according to Equation (3). The degree of inhibition (%) is expressed as 100 (1 − *k*/*k*_0_), where *k* and *k*_0_ are *k*_cat_/*K*_m_ of quinone in the presence and in the absence of NAD^+^, respectively. The *K*_d_ of the enzyme complex with NAD^+^, corresponding to the half-maximal degree of inhibition, were obtained by the fitting of dependence of the inhibition degree on NAD^+^ concentration to the parabolic expression using SigmaPlot 14 (Systat Software, Inc.).

The rates of enzymatic NADH oxidation in the presence of quinones, nitroaromatics, and benzylviologen were determined using the value ∆ε_340_ = 6.2 mM^−1^cm^−1^, and they were corrected for the intrinsic NADH-oxidase activity of *Pf*NDH2 (0.01 s^−1^). When 50 µM of cytochrome *c* was added to the reaction mixture, its quinone- and nitroaromatic-mediated reduction was assessed according to ∆ε_550_ = 20 mM^−1^cm^−1^. The ferricyanide reduction rate was measured according to ∆ε_420_ = 1.03 mM^−1^cm^−1^. The stock solutions of organic compounds were prepared in DMSO; the final concentration of DMSO in reaction mixtures was 1% (*v*/*v*). The concentrations of nitrite formed during the reduction of tetryl were determined spectrophotometrically at 540 nm, monitoring the formation of azo dye in the presence of sulfanilamide, naphthylethylene diamine dihydrochloride, and 4-fold diluted reaction mixture, as described [58]. The 10–200 µM NaNO_2_ solutions were used for the calibration curve.

### 4.4. Molecular Docking Studies

The molecular docking studies of *Pf*NDH2 chain A (PDB id: 5JWB) were performed using the GNINA program, v. 1.0 [43]. For the optimization, we used all default options in the GNINA software, v. 1.0 except for the scores used to rank poses during docking (“--pose_sort_order CNNaffinity”). The characteristics of GNINA and its application are presented in more detail in Supplementary Materials (S1).

The protein structure was prepared for docking using the ChimeraX program, v. 1.7.1 [59]. The compound 3D structures were taken from the PubChem database [60] and reoptimized using MMFF94s force field [61] within Avogadro viewer, v. 1.2.0 [62]. The conversion into PDBQT format suitable for docking was performed using AutoDockTools v. 1.5.6 [63] for the protein and OpenBabel, v. 3.1.1 [64] for the organic molecules. Root mean square deviations (RMSDs) were computed using the DockRMSD software, v. 1.1 [65]. The ligand–protein interface contact residue areas were computed using the Voronota software [66] (v. 1.29.4307). The reliability of the docking results was verified in the following way: (a) the docking data revealed the similarity between the binding mode and conformation of ubiquinone in its complex with *S. cerevisiae* Ndi1 and NAD(H) with the data of the X-ray crystallography of their ternary complex (PDB id: 7G43) (Supplementary Materials S1, Figure S1). This structure was chosen because it contains a ubiquinone ligand located in the proximity of the FAD/NAD(H) complex, which is the binding mode we were looking for; (b) the same procedure was repeated with the complex of *Pf*NDH2 and NAD(H) (PDB id: 5JWB), initially assuming that ubiquinone binds in the same conformation as in the X-ray structure of the ternary complex of Ndi1 (PDB id:7G43). Further docking details are presented in Supplementary Materials (S1).

## Figures and Tables

**Table 1:** The single-electron reduction midpoint potentials ($E_{7}^{1}$), octanol/water distribution coefficients at pH 7.0 (log *D*) of nonphysiological oxidants of *Pf*NDH2, and the steady-state rate constants of their reduction ($k_{\mathrm{cat}}^{\mathrm{app}}$ and *k*_cat_/*K*_m_) at [NADH] = 100 µM, 0.1 M Hepes with 0.15 M NaCl and 1.0 mM EDTA, pH 7.0, 25 °C. The values of $E_{7}^{1}$ are taken from References [35,36].

No.	Compound	$E_{7}^{1}$ (V)	log *D*	$k_{\mathrm{cat}}^{\mathrm{app}}$ (s^−1^)	*k*_cat_/*K*_m_ (M^−1^s^−1^)
**Quinones**					
**1**	1,4-Benzoquinone	0.09	1.02	14.00 ± 0.92	3.30 ± 0.21 × 10^5^
**2**	2-Methyl-1,4-benzoquinone	0.01	1.42	8.50 ± 0.87	2.10 ± 0.22 × 10^5^
**3**	2,5-Diaziridinyl-1,4-benzoquinone (DZQ)	−0.05	0.00	9.00 ± 0.81	3.72 ± 0.32 × 10^5^
**4**	2,6-Dimethyl-1,4-benzoquinone	−0.08	1.82	10.80 ± 0.71	2.85 ± 0.19 × 10^5^
**5**	5-Hydroxy-1,4-naphthoquinone (juglone)	−0.09	1.82	5.85 ± 0.32	8.11 ± 0.58 × 10^5^
**6**	5,8-Dihydroxy-1,4-naphthoquinone (naphthazarin)	−0.11	2.17	14.30 ± 1.01	6.95 ± 0.39 × 10^5^
**7**	9,10-Phenanthrene quinone	−0.12	2.92	15.55 ± 0.98	7.55 ± 0.43 × 10^5^
**8**	1,4-Naphthoquinone	−0.15	1.50	17.50 ± 1.05	5.45 ± 0.24 × 10^5^
**9**	2-Methyl-1,4-naphthoquinone (menadione)	−0.20	1.89	7.05 ± 0.25	9.25 ± 0.45 × 10^5^
**10**	Trimethyl-aziridinyl-1,4-benzoquinone	−0.23	1.70	20.90 ± 1.29	1.44 ± 0.11 × 10^5^
**11**	2,5-Dimethyl-3,6-diaziridinyl-1,4-benzoquinone (MeDZQ)	−0.23	0.79	1.60 ± 0.09	5.72 ± 0.31 × 10^4^
**12**	Ubiquinone (Q1)	−0.24	2.22	36.70 ± 2.23	1.78 ± 0.09 × 10^6^
**13**	Decylubiquinone	−0.24	5.00	32.10 ± 2.02	2.75 ± 0.19 × 10^6^
**14**	Idebenone	−0.24	3.57	41.00 ± 2.54	2.40 ± 0.16 × 10^6^
**15**	Tetramethyl-1,4-benzoquinone (duroquinone)	−0.26	2.61	13.00 ± 0.58	4.12 ± 0.26 × 10^5^
**16**	1,4-Dihydroxy-9,10-anthraquinone	−0.30	3.60	-	2.02 ± 0.14 × 10^3^
**17**	1,8-Dihydroxy-9,10-anthraquinone	−0.33	3.60	-	4.41 ± 0.27 × 10^3^
**18**	2-Hydroxy-1,4-naphthoquinone	−0.41	−0.70	0.42 ± 0.06	1.75 ± 0.19 × 10^4^
**Nitroaromatic compounds**					
**19**	2,4,6-Trinitrophenyl-*N*-methylnitramine (tetryl)	−0.191	1.38	7.40 ± 0.55	1.50 ± 0.11 × 10^5^
**20**	2,4,6-Trinitrotoluene	−0.253	2.31	2.40 ± 0.55	3.20 ± 0.19 × 10^4^
**21**	*p*-Dinitrobenzene	−0.255	1.85	7.35 ± 0.49	6.09 ± 0.29 × 10^4^
**22**	Nitrofurantoin	−0.255	−0.25	0.60 ± 0.05	2.25 ± 0.18 × 10^3^
**23**	Nifuroxime	−0.255	−0.35	1.30 ± 0.08	1.40 ± 0.11 × 10^4^
**24**	*o*-Dinitrobenzene	−0.287	1.85	1.10 ± 0.06	3.54 ± 0.15 × 10^3^
**25**	*p*-Nitrobenzaldehyde	−0.325	1.63	1.50 ± 0.07	9.95 ± 0.08 × 10^2^
**26**	*m*-Dinitrobenzene	−0.345	1.85	0.60 ± 0.04	3.37 ± 0.12 × 10^3^
**27**	*p*-Nitroacetophenone	−0.355	1.47	-	2.70 ± 0.13 × 10^2^
**28**	*p*-Nitrobenzoic acid	−0.425	−1.66	0.20 ± 0.02	1.79 ± 0.11 × 10^3^
**29**	Nitrobenzene	−0.485	1.91	0.80 ± 0.10	2.49 ± 0.20 × 10^2^
**Single-electron acceptors**					
**30**	Ferricyanide	0.410	-	6.80 ± 0.32	5.10 ± 0.24 × 10^4^
**31**	Benzylviologen	−0.354	-	0.50 ± 0.03	3.00 ± 0.16 × 10^3^

## Data Availability

The data are available from the corresponding author upon reasonable request.

## Supplementary Materials

The following supporting information can be downloaded at: https://www.mdpi.com/article/10.3390/ijms26062509/s1. Reference [67] is cited in the Supplementary Materials.
