# Supplementary material for: Reactions of Plasmodium falciparum Type II NADH: Ubiquinone Oxidoreductase with Nonphysiological Quinoidal and Nitroaromatic Oxidants
Source: Int J Mol Sci. 2025 Mar 11;26(6):2509. doi: 10.3390/ijms26062509 (PMC11941790; doi:10.3390/ijms26062509)
Supplement: Supplementary file 1 [file ijms-26-02509-s001.zip › ijms-3462910-supplementary.pdf]

Reactions of *Plasmodium falciparum* Type II NADH: ubiquinone Oxidoreductase with Nonphysiological Quinoidal and Nitroaromatic Oxidants

Lina Misevičienė, Marie-Pierre Golinelli-Cohen, Visvaldas Kairys, Audronė Marozienė, Mindaugas Lesanavičius and Narimantas Čėnas

Supplementary Materials

S1 – GNINA molecular docking protocol and its validation

GNINA calculates two docking parameters, Machine Learning based Convolutional Neural Network (CNN) scoring affinity (CNNaffinity), and the pose score (or CNNscore, i.e., similarity of the pose to the X-ray analysis geometry) which is a default option [1]. Initially, the program generates best 50 candidate poses that are later re-scored using one of the two Machine Learning scores. This approach is helpful in developing of the optimal docking protocol.

However, in some cases the default option (CNN score) prioritized the poses with relatively poor calculated affinities**.** For this reason, we used the double scoring approach. For example, analyzing the candidate poses of ubiquinone bound to *S. cerevisiae* Ndi1 (PDB id: 4G73), the re-scoring using CNN affinity identified two poses, ranked 1 and 2, the later being the correct binding mode available from the X-ray data [2]. However, its affinity was negligibly less (by 0.002 pKa units) compared to rank 1 pose, but the pose score (0.90) was much better compared to rank 1 pose (0.78). Therefore, when choosing the 'decisive' conformation, the approach was to rank poses using CNNaffinity ("--pose_sort_order CNNaffinity" option in GNINA), then choose rank 1 pose, unless there was a high ranked pose with a better pose score (CNNscore). This double scoring approach seemed to be superior to a single scoring approach. For comparison, we also have tried Smina docking program [3] to dock ubiquinone into 4G73, and the correct binding mode was ranked 3. It should be noted that for the most cases presented in this paper this protocol picked the top ranked pose as the best one, except for TNT and ubiquinone-1 (pose ranked 2).

The docking by GNINA was validated by docking ubiquinone into 4G73 (chain A) [1]and *Pf*NDH2 (PDB id: 5JWB, chain A). As described above, we chose rank 2 pose as the best one taking into account its better pose score. In this case, Root Mean Square Deviation (RMSD) of ubiquinone quinone ring for the selected best pose was reasonable, 1.671 Å, especially having in mind that the most important H-bonding was correctly detected by the docking (Figure S1A). Docking ubiquinone into *Pf*NDH2 lead to a better RMSD value, 1.381 Å (Figure S1B). The same binding mode was successfully predicted for the docked quinones with the protocol detailed above (see Figure 7 in the manuscript), therefore the same protocol was also used for nitroaromatic compounds (Figure 8). For quantitative evaluation of ligand-amino acid interactions, the contact areas and the nearest distances between ligand and the residues were calculated using Voronota software [4] (Table S1).

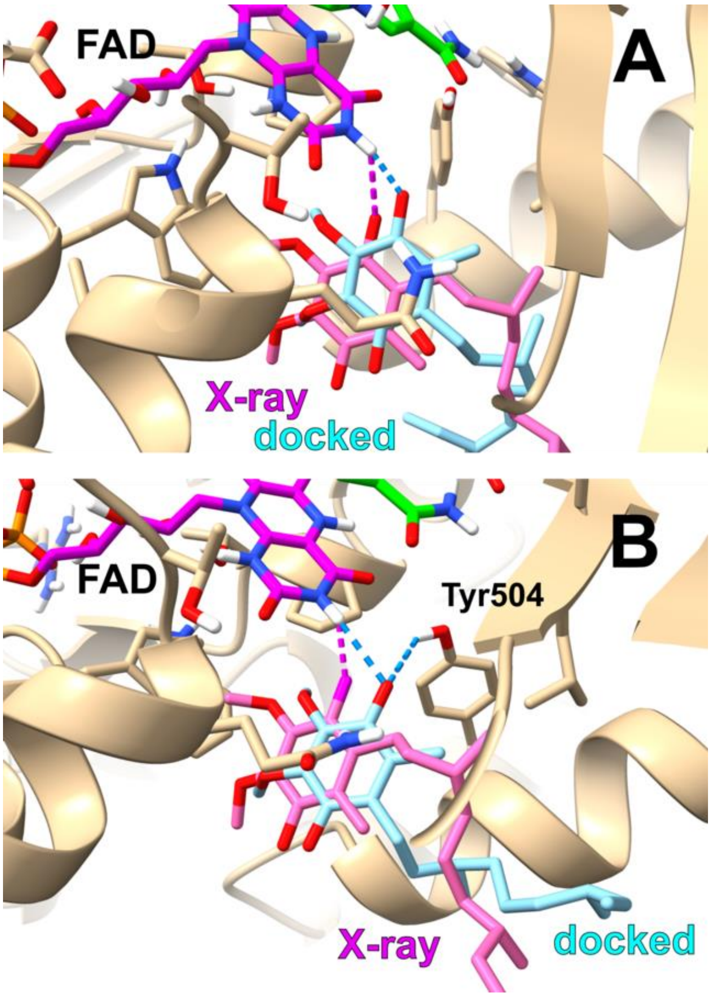

Figure S1. The docking of ubiquinone into the active centers of *S. cerevisiae* Ndi1 (PDB id: 4G73) [2] **(A)** and *Pf*NDH2 (PDB id: 5JWB) **(B).** The docked position of ubiquinone is shown in light blue, and that obtained from the X-ray analysis of its complex with Ndi1 is shown in pink, including its superposition on the structure of *Pf*NDH2. A part of FAD is shown in magenta, a part of nicotinamide ring of NAD(H) is shown in green. The pink and light blue dashed lines denote hydrogen bonds for the X-ray and docked conformation, correspondingly.

Table S1. The contact areas and the nearest distances between the docked ligands and the surrounding residues in *Pf*NDH2 calculated using Voronota software [4].

Residue[a]	Area, Å^2	Minimal distance, Å	Notes	Residue[a]	Area, Å^2	Minimal distance, Å	Notes
**duroquinone**				**menadione**			
Trp50	18.9	3.41		Trp50	15.8	3.71	
Phe77	1.0	5.57		Phe77	0.7	5.89	
Pro79	9.9	3.76		Pro79	8.1	4.41	

Ala436	12.3	3.23		Ala436	10.0	3.62	
Gln437	39.9	3.37		Gln437	38.1	3.17	
Lys440	20.3	3.82		Lys440	14.3	4.33	
Gln441	0.4	5.60		Lys470	23.7	3.85	
Lys470	21.6	3.83		Gly471	9.0	3.64	
Gly471	7.1	3.91		Ser472	3.2	5.04	
Ser472	2.5	5.17		Leu473	18.0	3.75	
Leu473	14.2	3.87		Leu485	4.1	5.11	
Val503	4.7	4.81		Val503	8.5	4.64	
Tyr504	11.2	3.71	H-bond	Tyr504	13.5	3.05	H-bond
Leu507	43.6	3.32		Leu507	41.6	3.51	
FAD601	19.4	3.12	H-bond	FAD601	19.4	3.09	H-bond
NAD608	1.4	4.90		NAD608	2.9	4.37	
Total	135.3			*Total*	107.9		
**ubiquinone-1**				**decylubiquinone**			
Trp50	19.0	3.70		Trp50	18.9	3.66	
Phe77	2.0	5.12		Phe77	1.8	5.18	
Pro79	9.7	3.62		Pro79	9.7	3.66	
Ala436	10.1	3.50		Ala436	9.9	3.53	
Gln437	39.4	3.18		Gln437	39.1	3.13	
Lys440	20.4	3.50		Lys440	24.5	3.45	
Lys470	36.8	3.66		Lys470	34.5	3.69	
Gly471	8.4	3.47		Gly471	8.7	3.42	
Ser472	4.3	4.46		Ser472	4.6	4.36	
Leu473	17.2	3.24		Leu473	17.6	3.15	
Leu485	0.6	5.98		Leu485	1.5	5.90	
Val503	24.8	3.60		Val503	30.5	3.34	
Tyr504	11.7	3.94	H-bond	Tyr504	11.5	3.98	H-bond
Gln506	10.9	4.98		Gln506	44.5	3.43	
Leu507	59.0	3.33		Leu507	67.8	3.49	
FAD601	19.8	3.01	H-bond	FAD601	19.9	2.96	H-bond
NAD608	2.6	4.60		NAD608	2.9	4.51	
Total	159.4			*Total*	183.7		
**idebenone**				**trinitrotoluene**			
Trp50	16.6	3.38		Trp50	20.0	3.31	
Phe77	1.9	5.12		Phe77	0.7	5.82	
Pro79	8.8	4.03		Pro79	8.4	4.20	
Ala436	9.7	3.62		Ala436	11.5	3.30	
Gln437	43.4	3.11		Gln437	42.6	3.01	H-bond
Lys440	22.1	3.51		Lys440	23.1	3.49	H-bond
Gln441	6.8	4.85		Gln441	0.1	5.52	
Lys470	37.9	3.58		Lys470	33.3	3.41	H-bond

Gly471	8.6	3.48		Gly471	7.0	3.96	
Ser472	4.3	4.51		Ser472	6.0	4.74	
Leu473	20.9	3.46		Leu473	13.9	4.41	
Leu485	17.2	3.66		Val503	5.6	5.28	
Phe499	21.2	3.61		Tyr504	13.0	3.12	H-bond
Val502	13.8	3.60		Leu507	51.1	3.24	
Val503	34.3	3.28		FAD601	23.2	3.03	H-bond
Tyr504	10.5	4.46	H-bond	NAD608	5.1	4.15	
Gln506	20.4	3.01	H-bond	*Total*	192.0		
Leu507	49.4	3.32					
FAD601	18.3	2.97	H-bond				
NAD608	2.1	4.91					
Total	211.4						
**tetryl**							
Trp50	14.4	3.40					
Phe77	1.6	5.04					
Pro79	8.3	4.58					
Thr435	0.2	5.48					
Ala436	8.6	3.34					
Gln437	46.6	2.79	H-bond				
Lys440	23.6	3.96	H-bond				
Gln441	6.8	4.11	H-bond				
Lys470	39.2	2.94	H-bond				
Gly471	11.2	2.97					
Ser472	8.8	3.51					
Leu473	21.3	3.51	H-bond				
Leu485	2.4	5.07					
Val503	10.3	3.43					
Tyr504	15.4	3.21					
Leu507	49.6	3.26					
FAD601	24.7	3.00	H-bond				
NAD608	5.6	4.07					
Total	172.6						

S2 - Derivation of steady-state rate equations of *Pf*NDH2

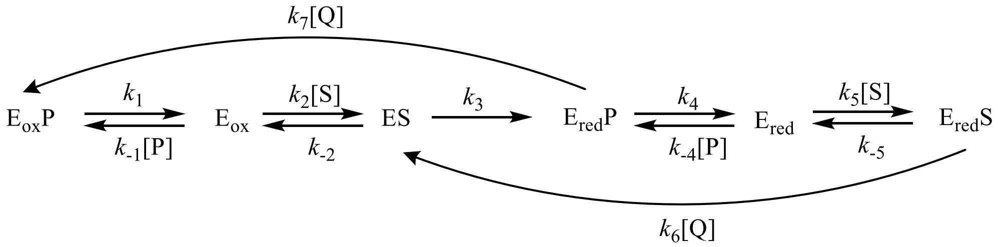

(Scheme S1)

We assume that the enzyme redox forms and complexes E_{red}, $E_{red}P$ and $E_{red}S$ (Scheme S1) are in rapid equilibrium, and at high S and/or P concentrations, $[E_{red}] \cong 0$. The K_d of complexes of complexes $E_{ox}P$, $E_{red}P$ and $E_{red}S$ are expressed as equilibrium constants $K_1 = k_1/k_{-1}$, $K_4 = k_4/k_{-4}$, and $K_5 = k_{-5}/k_5$, respectively. Using Cha's simplification [5], one can multiply the rate constants k_6 and k_7 by fractions f_1 and f_2, which are expressed as:

$$f_1 = f(E_{red}S) = [E_{red}S]/([E_{red}S] + [E_{red}P]) = ([S]/K_5)/([S]/K_5 + [P]/K_4), \qquad \text{(S2-1)}$$

and

$$f_2 = f(E_{red}P) = [E_{red}P]/([E_{red}S] + [E_{red}P]) = ([P]/K_4)/([S]/K_5 + [P]/K_4). \qquad \text{(S2- 2)}$$

Subsequently, Scheme S1 may be converted into Scheme S2:

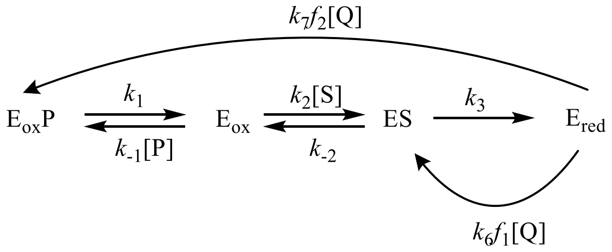

(Scheme S2)

The total enzyme concentration is equal to the sum of concentration of its redox forms and complexes:

$$[E] = [E_{ox}] + [E_{ox}P] + [ES] + [E_{red}], \qquad \text{(S2-3)}$$

and the reaction rate (v) may be expressed as:

$$v = k_3[ES]. \qquad \text{(S2-4)}$$

Under steady-state conditions,

$$d[E_{ox}]/dt = k_1[E_{ox}P] – (k_{-1}[P] + k_2[S])[E_{ox}] + k_{-2}[ES] = 0, \qquad \text{(S2-5)}$$

$$d[E_{ox}P]/dt = k_{-1}[E_{ox}][P] + k_7f_2 [E_{red}][Q] – k_1[E_{ox}P] = 0, \qquad \text{(S2-6)}$$

$$d[ES]/dt = k_2[E_{ox}][S] + k_6f_1 [E_{red}][Q] – (k_{-2} + k_3[E_S] = 0, \qquad \text{(S2-7)}$$

and

$$d[E_{red}]/dt = k_3[ES] – (k_6f_1[P] + k_7f_2)[E_{red}][Q] = 0 \qquad \text{(S2-8).}$$

Further, with the use of Equations (S2-5 – S2-8), the concentrations $[E_{ox}]$, $[E_{ox}P]$ and $[E_{red}]$ are expressed in terms of [ES], and they are inserted into Equation (S2-3). Next, the dividing Equation (S2-3) by Equation (S2-4) gives the final rate expression (Equation S2-9):

$$\frac{[E]}{v} = \frac{1}{k_3}\left(1+\frac{k_{-2}}{k_2[S]}\left(1+\frac{[P]}{K_1}\right)\right)+\frac{K_5[P]+K_4[S]}{k_7[P]K_5+k_6[S]K_4}\left(\frac{1}{[Q]}+\frac{k_7[P]K_5}{k_2[S](K_5[P]+K_4[S])}\left(1+\frac{[P]}{K_1}\right)\right) \quad \text{(S9)}$$

References:

1. McNutt, A.T.; Francoeur, P.; Aggarwal R.; Masuda, T.; Meli, R.; Ragoza, M.; Sunseri, J.; Koes, D.R. GNINA1:0: molecular docking with deap learning. *J. Cheminform.* **2021,** *13,* 43.
2. Feng, Y.; Li, W.; Li, J.; Wang, J.; Ge, J.; Xu, D.; Liu, Y.; Wu, K.; Zheng, Q.; Wu, J-W.; et al. Structural insights into the type-II mitochondrial NADH dehydrogenases. *Nature* **2012,** *491*, 478-482.
3. Koes, D.R.; Baumgartner, M.P.; Camacho, C.J. Lesons learned in empirical scoring with smina from the CSAR 2011. *J. Chem. Inf. Model.* **2013,** *53,* 1893-1904.
4. Olechnovič, K.; Venclovas, Č. Voronota: A fast and reliable tool for computing the vertices of the Voronoi diagram of atomic balls. *J. Comput. Chem.* **2014,** 35, 672-681.
5. Huang C.Y. Derivation and initial velocity and isotope exchange rate equations. *Methods Enzymol.* **1979,** *63,* 54-84.
